# Early Detection of Cardiovascular Changes After Radiotherapy for Breast Cancer: Protocol for a European Multicenter Prospective Cohort Study (MEDIRAD EARLY HEART Study)

**DOI:** 10.2196/resprot.9906

**Published:** 2018-10-01

**Authors:** Valentin Walker, Anne Crijns, Johannes Langendijk, Daan Spoor, Rozemarijn Vliegenthart, Stephanie E Combs, Michael Mayinger, Arantxa Eraso, Ferran Guedea, Manuela Fiuza, Susana Constantino, Radia Tamarat, Dominique Laurier, Jean Ferrières, Elie Mousseaux, Elisabeth Cardis, Sophie Jacob

**Affiliations:** 1 Pôle Santé-Environnement (PSE-SANTE), Service de recherche sur les effets biologiques et sanitaires des rayonnements ionisants (SESANE) Laboratoire d'épidémiologie des rayonnements ionisants (LEPID) Institut de Radioprotection et de Sûreté Nucléaire (IRSN) Fontenay-aux-Roses France; 2 Department of Radiation Oncology University Medical Center Groningen University of Groningen Groningen Netherlands; 3 Center for Medical Imaging, Department of Radiology University Medical Center Groningen University of Groningen Groningen Netherlands; 4 Department of Radiation Oncology Technische Universität München (TUM) München Germany; 5 Department of Radiation Sciences (DRS) Institute of Innovative Radiotherapy (iRT) Helmholtz Zentrum München (HMGU) München Germany; 6 Deutsches Konsortium für Translationale Krebsforschung (DKTK) Partner Site Munich München Germany; 7 Department of Radiation Oncology Institut Català d'Oncologia Girona Spain; 8 Department of Radiation Oncology Institut Català d'Oncologia L’Hospitalet del Llobregat Spain; 9 Department of Cardiology Centro Cardiovascular da Universidade de Lisboa Lisbon Portugal; 10 Laboratory of Angiogenesis Centro Cardiovascular da Universidade de Lisboa Lisbon Portugal; 11 Pôle Santé-Environnement (PSE-SANTE) Institut de Radioprotection et de Sûreté Nucléaire (IRSN) Fontenay-aux-Roses France; 12 Pôle Santé-Environnement (PSE-SANTE), Service de recherche sur les effets biologiques et sanitaires des rayonnements ionisants (SESANE) Institut de Radioprotection et de Sûreté Nucléaire (IRSN) Fontenay-aux-Roses France; 13 Department of Cardiology B and Epidemiology University Hospital Toulouse France; 14 Unite Mixte de Recherche (UMR) 1027 The Institut national de la santé et de la recherche médicale (INSERM) Toulouse France; 15 Department of Radiology Hôpital Européen Georges Pompidou Paris Descartes University Paris France; 16 Institute for Global Health (ISGlobal) Radiation Programme Barcelona Biomedical Research Park (PRBB) Barcelona Spain; 17 Pompeu Fabra University (UPF) Barcelona Spain; 18 Consorcio Centro de Investigación Biomédica en Red Epidemiologia y Salud Pública (CIBERESP) Madrid Spain

**Keywords:** biomarkers, breast cancer, cardiotoxicity, cardiac diagnostic imaging, radiotherapy, radiation dosimetry

## Abstract

**Background:**

Breast cancer is the most common cancer among women, and radiotherapy plays a major role in its treatment. However, breast cancer radiotherapy can lead to incidental irradiation of the heart, resulting in an increased risk for a variety of heart diseases arising many years after radiotherapy. Therefore, identifying breast cancer patients at the highest risk for radiation-induced cardiac complications is crucial for developing strategies for primary and secondary prevention, which may contribute to healthy aging. There is still a need for precise knowledge on the relationship between radiation dose to specific cardiac structures and early subclinical cardiac changes and their occurrence over time that could finally lead to cardiac complications.

**Objective:**

The MEDIRAD EARLY HEART study aims to identify and validate new cardiac imaging and circulating biomarkers of radiation-induced cardiovascular changes arising within first 2 years of breast cancer radiotherapy and to develop risk models integrating these biomarkers combined with precise dose metrics of cardiac structures based on three-dimensional dosimetry.

**Methods:**

The EARLY HEART study is a multicenter, prospective cohort study in which 250 women treated for breast cancer and followed for 2 years after radiotherapy will be included. Women treated with radiotherapy without chemotherapy for a unilateral breast cancer and aged 40-75 years meet the inclusion criteria. Baseline and follow-up data include cardiac measurements based on two-dimensional speckle-tracking echocardiography, computed tomography coronary angiography, cardiac magnetic resonance imaging, and a wide panel of circulating biomarkers of cardiac injury. The absorbed dose will be evaluated globally for the heart and different substructures. Furthermore, the dose-response relationship will allow modeling the radiation-induced occurrence and evolution of subclinical cardiac lesions and biomarkers to develop prediction models.

**Results:**

This study details the protocol of the MEDIRAD EARLY HEART study and presents the main limits and advantages of this international project. The inclusion of patients began in 2017. Preliminary results are expected to be published in 2019, and complete analysis should be published in 2021.

**Conclusions:**

The MEDIRAD EARLY HEART study will allow identifying the main cardiac imaging and blood-based determinants of radiation-induced cardiac injuries to better propose primary and secondary preventive measures in order to contribute to enhanced patient care and quality of life.

**Trial Registration:**

ClinicalTrials.gov NCT03297346; https://clinicaltrials.gov/ct2/show/NCT03297346 (Archived by WebCite at http://www.webcitation.org/72KS7MIUU)

**Registered Report Identifier:**

RR1-10.2196/9906

## Introduction

Cancer is a major burden on society, which is expected to increase worldwide due to the growth and aging of the population. Furthermore, breast cancer (BC) is the most commonly diagnosed cancer among women. In 2016, BC accounted for 29% of the total new cancer cases and 14% of the total cancer-related deaths among women worldwide [1]. While its incidence has been increasing in the past decade, the prognosis has markedly improved over the last decades with enhanced screening and medical support for these patients, resulting in longer life expectancy and 5-year relative survival rate > 60%-95% according to the age class in most Western countries [2]. This is due to improved detection programs and treatment modalities, including improved radiotherapy (RT) techniques. In the past few decades, RT has been increasingly used for the treatment of BC. Research has shown that RT presents a benefit in terms of reducing local recurrence and deaths resulting from BC [3]. Due to this increased survival rate, the interest in potential adverse effects and long-term consequences related to RT has spiked.

RT plays a major role in the treatment of BC, as >60% of all BC patients are irradiated as part of their curative treatment, and it improves outcome in terms of reducing the local recurrence and deaths resulting from BC [4]. However, BC RT can lead to incidental irradiation of the heart due to its presence within the irradiation field, resulting in an increased risk for a variety of heart diseases arising many years after RT [5], such as ischemic heart disease, congestive heart failure, arrhythmias, conduction defects, valvular disease, or pericarditis, with relative risks within the range of 1.2-3.5 on comparing patients with left-sided BC (corresponding to patients with higher exposure to the heart) to patients with right-sided BC [6-8]. In parallel with the increase in the incidence of BC, the prevalence of BC survivors at risk for cardiac complications will, thus, gradually increase [3,4,6-8]. Technological developments in RT over the last decades, such as intensity-modulated radiotherapy or volumetric modulated arc therapy and deep inspiration breath-hold, have allowed for a marked reduction of cardiac doses, in particular for patients with left-sided BC; the mean heart dose has decreased from >5 Gy in the 1950s to <3 Gy in the last decade [9]. It has been shown that the use of these techniques in BC patients is of concern to the radiation oncologists and is widely implemented [10,11]. However, cardiac damage has been shown to be correlated with the mean heart dose, with a 7.4% increase in the rate of acute coronary events per 1 Gy (95% CI 2.9-14.5; *P*<.001), with no minimum threshold for risk [8]. The risk for acute coronary events within first 9 years of RT has recently been confirmed by an additional publication [12], showing marked relationship between cardiac radiation dose and acute coronary event incidence. Therefore, as there appears to be no threshold dose below which cardiac complications do not appear, radiation-induced cardiac diseases remain potential, severe, late complications of BC RT. Moreover, dose distributions in the heart are extremely heterogeneous, and some cardiac substructures such as the apex and the apical-anterior segment can still receive higher doses. Therefore, there is still an urgent need for preventive measures. Long before the onset of clinically significant late cardiac complications, subclinical cardiac changes may occur over weeks, months, or years after RT that can be detected using anatomical and functional cardiac imaging or circulating biomarkers [13].

In the context of BC RT-induced functional cardiac changes, a recent advanced echocardiographic technique (automated two-dimensional speckle-tracking echocardiography, ECHO-ST, or cardiac strain] has been used for detecting and quantifying subtle (subclinical) disturbances in left ventricular strain and function. A Belgian team first showed that global longitudinal strain and strain rate were substantially decreased (mean 5%) during the first year following breast RT [14]. This result was confirmed by other studies in patients with left-sided BC whose global longitudinal strain and apical strain were diminished. In addition, the basal regions showed a compensatory increase in function, although not sufficient to compensate for the global functional loss resulting in a decrease in the global longitudinal strain [14-18]. Moreover, among patients with right-sided BC RT, a deterioration in basal anterior strain segments after RT was observed, whereas the global function remained unaffected [19].

Cardiac computed tomography (CT) without contrast and CT coronary angiography (CTCA) allows the determination of the coronary artery calcium (CAC) score and detection of plaques and stenosis along the coronary arteries. The presence and diffusion of calcium, plaque, and stenosis expose patients to a higher risk for coronary artery disease (CAD). Based on an analysis of 15 segments of coronary arteries per patient with acute chest pain, an increase in calcified and noncalcified plaques of around 15% was observed during a 2-year follow-up [20]. While this study did not consider radiation exposure of the heart, it does illustrate that the CTCA could be used for monitoring short-term coronary changes and has the potential to detect the onset or progression of early coronary changes due to irradiation among women treated with BC RT. To date, three studies have measured the amount of CAC in the years following RT treatment for BC. In two studies, no elevated CAC scores in BC patients were found 5-15.7 years after RT treatment, whereas one study did find an increase in the CAC score depending on the radiation dose to the heart [21-23]. Among the studies that did not find a CAC score increase, one did not include baseline CAC scores and the other only included a relatively small number of patients, making it difficult to draw definitive conclusions from these two studies. In young Hodgkin’s lymphoma survivors (all aged <55 years), elevated CAC scores have been found 5-35 years after RT [24-26]. A study concerning the general population investigated CAC scores at baseline and after 10 years of follow-up [27]; the results showed that the diagnosis of cancer and its treatments were markedly associated with an increase in CAC scores even after accounting for cardiac risk factors. The results of these studies suggest that RT is associated with increased CAC scores in the long term and, therefore, support the hypothesis that accelerated atherosclerosis is one of the mechanisms contributing to an increase in RT-induced cardiac events after cardiac irradiation.

Cardiac magnetic resonance imaging (MRI), which is considered as the gold standard to characterize myocardial tissue and measure ventricular volumes and function, was used to show that right ventricular systolic function was decreased in a BC cohort at 24 months after RT [16]. Furthermore, a decline in temporary ejection fraction was observed on MRI (in patients treated with three-dimensional [3D] conformal radiotherapy and not in patients treated with intensity-modulated radiotherapy) at 6 months, which resolved at 24 months. Left and right ventricular systolic functions determined using MRI were reduced at 24 months (but still within the normal range) for the whole cohort. Furthermore, using T1 mapping, a promising technique to quantify morphologic tissue injuries, it was shown in cancer survivors (including BC patients) that interstitial or diffuse myocardial fibrosis was elevated 3 years after anthracycline-based chemotherapy independent of the underlying cancer or comorbidities, suggesting that imaging biomarkers of interstitial fibrosis are related to prior receipt of potentially cardiotoxic cancer treatment [28], which may also include ionizing radiation.

A major challenge is the identification of reliable circulating biomarkers that could help diagnose and predict cardiotoxicity. Many classical biomarkers (eg, C-reactive protein, N-terminal pro-B-type natriuretic peptide, and troponin) have been shown to be potential biomarkers for cardiac damage after RT [29]. In addition, circulating inflammatory cytokines indicated tissue inflammation [30]. Radiation injury to the myocardium is primarily caused by damage to the microvasculature, leading to inflammatory and thrombotic changes, capillary loss, focal ischemia, and interstitial fibrosis [31,32]; these pathological changes can cause congestive heart failure.

One relatively recent advancement in this area is the discovery of circulating extracellular vesicles (EVs), including microparticles and exosomes, and their emergence as mediators of a new important mechanism of cell-to-cell communication [33]. The role of EVs in mediating vascular dysfunction suggests that they may represent novel pathways in short- or long-distance paracrine intercellular signaling in the vascular environment. High levels of circulating EVs (involving microparticles) found in many cardiovascular diseases demonstrate the importance of platelet, monocyte, and endothelial activation and could condition remote sustainability illnesses [34].

Exosomes carry a composite cargo of cardiac microRNAs (miRNAs). MiRNAs are posttranscriptional inhibitory regulators of gene expression that are emerging as important mediators of intercellular communication, being involved in the transmission of biological signals between cells. To date, several miRNAs have been identified as having a primary impact on many biological processes that are of direct relevance to cardiovascular complications. The exosomal trafficking of miRNAs from the heart is largely unexplored. Interventional cardiologists have already provided evidence that cardiac-expressed miRNAs (miR-1, miR-133a, miR-133b, miR-208a, miR-208b, and miR-499) increase in the blood acutely following a myocardial infarction, and some of these studies have additionally scrutinized the diagnostic potential of miRNAs by comparing them with cardiac troponin [35,36]. In a clinical study, it has been demonstrated that the plasma concentration of EVs and their cargo of cardiac miRNAs increased in patients undergoing coronary artery bypass graft and were positively correlated with cardiac troponin [36]. Another hypothesis that remains to be explored is that the irradiation may be responsible for an increase in the number of circulating levels of EVs associated with certain miRNAs expressed by the cells of the heart tissue. Finally, studies have examined the global DNA methylation and risk for cardiovascular disease. The methylation of Bcl-2/adenovirus E1B 19kD-interacting protein 3, SuperOxyde Dismutase, Glutathione S-transferase P, Apolipoprotein E, B-cell lymphoma 2, BCL-2-associated X protein, and tissue inhibitor of metalloproteinases-3 promoters has been associated with the presence of CAD, which is the most prevalent cardiovascular disease following the exposure to radiation and may be useful for diagnostic and monitoring purposes [37].

These previous studies have shown that early subclinical cardiac changes can occur in BC patients after RT. Additionally, classical cardiac biomarkers have been shown to be potential candidates to monitor cardiac damage after RT. Identifying the main cardiac imaging and blood-based determinants of radiation-induced cardiac injuries is crucial for developing strategies for primary and secondary prevention. Primary prevention includes radiation dose optimization to the most critical structures of the heart. Secondary prevention requires identification of patients at risk as early as possible before and after RT. So far, little has been done regarding the relationship between dose distribution to different anatomical cardiac structures during RT and early cardiovascular changes that may lead to cardiac complications. Mathematical models have been developed in recent years with the aim of using dosimetric data to estimate a complication probability—the Normal Tissue Complication Probability (NTCP) models. Research on parameters to optimize the NTCP models (physical imaging, dosimetry, and clinical) is a major challenge to improve knowledge on the relationship between a received dose and toxicity to healthy tissue [38,39]. Moreover, the enrichment of these models with biological data is fundamental to improve risk prediction. Primary and secondary prevention measures require precise knowledge of the relationship between the dose to specific cardiac structures and the occurrence of early subclinical cardiac changes over time. To date, little has been done on elucidating the specific relationships between doses to cardiac structures and subsequent early cardiac toxicity, and NTCP models have been poorly exploited [40]. However, a prerequisite for further sparing of tissue subregions, as well as the analysis of NTCP properties, requires prospective identification of these structures, and their dose-volume properties must be taken into account in prospective treatment planning [41].

In this context, in the frame of the European MEDIRAD project [42], the EARLY HEART study was launched in July 2017 with the aims of identifying and validating the most important cardiac imaging biomarkers (based on ECHO-ST, CTCA, and MRI) and circulating biomarkers of radiation-induced cardiovascular changes arising within first 2 years of BC RT and of developing risk prediction models, such as NTCP models, integrating these biomarkers combined with precise dose metrics of cardiac structures based on 3D-dosimetry.

## Methods

### Study Design

MEDIRAD EARLY HEART (NCT03297346) is a multicenter, prospective cohort study that will include female patients with left- and right-sided BC treated with postoperative RT without chemotherapy after primary breast-conserving surgery. The patients will be followed for 2 years after RT, based on cardiac imaging and circulating biomarkers further detailed below. Five investigating centers are involved in the study: Clinique Pasteur (Toulouse, France) for the Institute of Radiation Protection and Nuclear Safety (Fontenay-aux-Roses, France), the University Medical Center Groningen (UMCG; Groningen, the Netherlands), die Klinikum rechts der Isar der Technischen Universität München (TUM MED; Munich, Germany), Institut Català d’Oncologia (Girona, Spain), and Centro Cardiovascular da Universidade de Lisboa (Lisbon, Portugal). Furthermore, Paris Descartes University (Paris, France) is involved in the study as core lab for the centralized analysis of cardiac CT and cardiac MRI. Data collection is expected to be performed until autumn 2020.

### Ethical Considerations

This study will be conducted in accordance with the Declaration of Helsinki (amended at the 64th World Medical Association General Assembly, Fortaleza, Brazil, October 2013) and in accordance with the principles of “Good Clinical Practice” and the Medical Research Involving Human Subjects Act (WMO). The 5 investigating centers have received approvals from their local ethical committees (France: Comité de Protection de Personnes Sud-Ouest IV, ID: CPP2015/66/2015-A00990-69–R1, and Agence Nationale de Sécurité des Médicaments, ID: 150873B-12; the Netherlands: Medisch Ethische Toetsingscommissie van het Universitair Medisch Centrum Groningen [METc UMCG], ID: METc 2017/379, NL62360.042.17; Germany: Ethikkomission der Technischen Universität München, ID: 235/17 S; Spain: Comitè d’Etica d’Investigatio CEAi GIRONA, ID: EARLY HEART v1.1 05/07/2017 i FIP v1.3; Portugal: Comissao de Ética do Centro Hospitalar Lisboa Norte e do Centro Académico de Medicina de Lisboa [CHLN e CAML], ID: 257/2017).

### Study Population, Site Participation, and Recruitment

In this study, we plan to include 250 female patients with unilateral BC treated at the 5 participating centers with postoperative modern photon-based planning CT after breast-conserving surgery, without chemotherapy, who are aged 40-75 years at the time of RT. The determination of the size of the study population is based on power calculation detailed later.

Textbox 1 details the additional inclusion and exclusion criteria as well as early dismissal (after inclusion) criteria. Each patient is included at the baseline before RT and followed for 2 years after RT. Informed consent of each patient was collected before inclusion.

**Inclusion, exclusion, and early dismissal criteria in the EARLY HEART study.**

Inclusion criteria

- Female unilateral breast cancer patients
- Treated with primary breast-conserving surgery for stage I-III invasive adenocarcinoma of the breast or ductal carcinoma *in situ*
- Age 40-75 years at the time of starting radiotherapy
- World Health Organization performance status 0-1
- Planned for radiotherapy alone to the breast with or without the lymph node areas
- Radiotherapy based on planning-computed tomography using either three-dimensional conformal radiotherapy, intensity-modulated radiotherapy, or volumetric modulated arc therapy/RapidArc
- Written informed consent

Exclusion criteria

- Male breast cancer patients
- Neoadjuvant or adjuvant chemotherapy
- M1 disease (metastatic breast cancer)
- Medical history of coronary artery disease and myocardial infarction and atrial fibrillation
- Previous thoracic or mediastinal radiation
- Contraindications to injection of iodinated contrast such as allergy or renal failure
- Pregnancy or lactation

Dismissal criteria

- Atrial fibrillation detected on electrocardiogram before radiotherapy
- Abnormal echocardiography before radiotherapy defined as either left ventricular ejection fraction <50% or longitudinal strain ≤−16% or longitudinal strain rate <−1% or abnormal wall motion
- Computed tomography coronary angiography results before radiotherapy requiring revascularization
- Presence of myocardial infarction based on magnetic resonance imaging before radiotherapy

### Conduct of the Study

#### Before Inclusion

At each center, radiation oncologists enable first contact with women diagnosed with BC. During the first visit with the radiation oncologist, he or she presents the study and its implications to female patients for whom RT is planned. The radiation oncologist ensures that women meet the inclusion criteria in the study and study information leaflet detailing the protocol is given with a consent form.

#### Before Start of Radiotherapy

About 1-2 weeks before the start of RT, women agreeing to participate in the study hand their signed written informed consent form and are then considered as included. Once included, the women’s follow-up protocol is implemented. Before the start of RT, several interventions are performed.

- An electrocardiogram to detect any arrhythmia, followed by an automated ECHO-ST, which is the most commonly used modality to evaluate myocardial dysfunction and a new technique for assessing myocardial deformation.
- CTCA using both low-dose, nonenhanced and enhanced CT scans to evaluate coronary artery lesions by assessing morphological information, including plaques and stenosis of the arteries, and determination of the CAC score.
- MRI to measure the ventricular function and size and wall thickness of the two heart chambers; tissue characterization by delayed enhancement; and pre- or postcontrast T1 mapping and precontrast T2 mapping of the left ventricle.
- Blood sampling (BLOOD) will be performed to allow analyses of circulating biomarkers based on a panel of circulating classical and novel blood-based biomarkers (further details below).

Furthermore, toxicity is assessed according to the Common Terminology Criteria for Adverse Events (version 4.03), which is widely accepted throughout the oncology community as the standard classification and severity grading scale for adverse events in cancer therapy clinical trials and other oncology settings.

#### Subsequent Follow-Up

**Figure 1:**
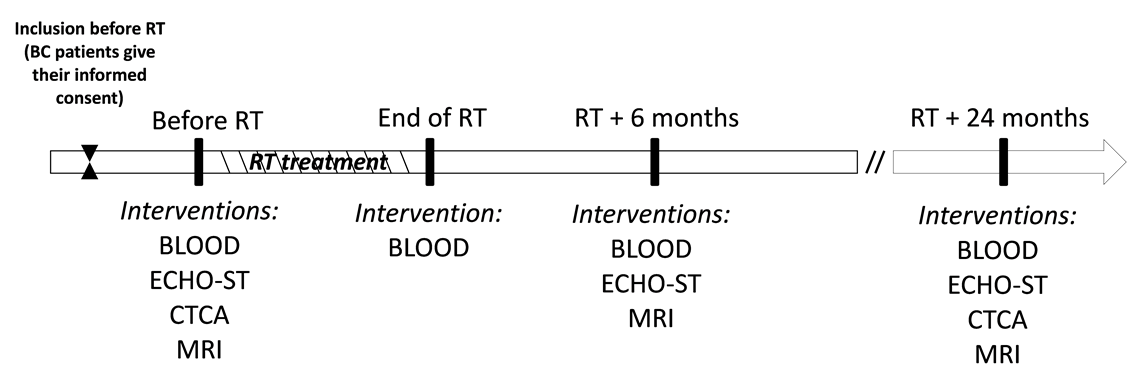
Main steps of the EARLY HEART study protocol. RT: radiotherapy; BC: breast cancer; BLOOD: blood sample for circulating biomarkers; ECHO-ST: two-dimensional speckle-tracking echocardiography; CTCA: computed tomography coronary angiography; MRI: magnetic resonance imaging.

During subsequent follow-up, imaging and circulating biomarkers are collected at specific time points after RT up to 24 months: (1) at the end of RT: BLOOD; (2) 6 months after RT: ECHO-ST, MRI, and BLOOD; (3) 2 years after RT: electrocardiogram and ECHO-ST, CTCA, MRI, and BLOOD. Figure 1 describes the different steps of the EARLY HEART study protocol.

### Data Collection

**Figure 2:**
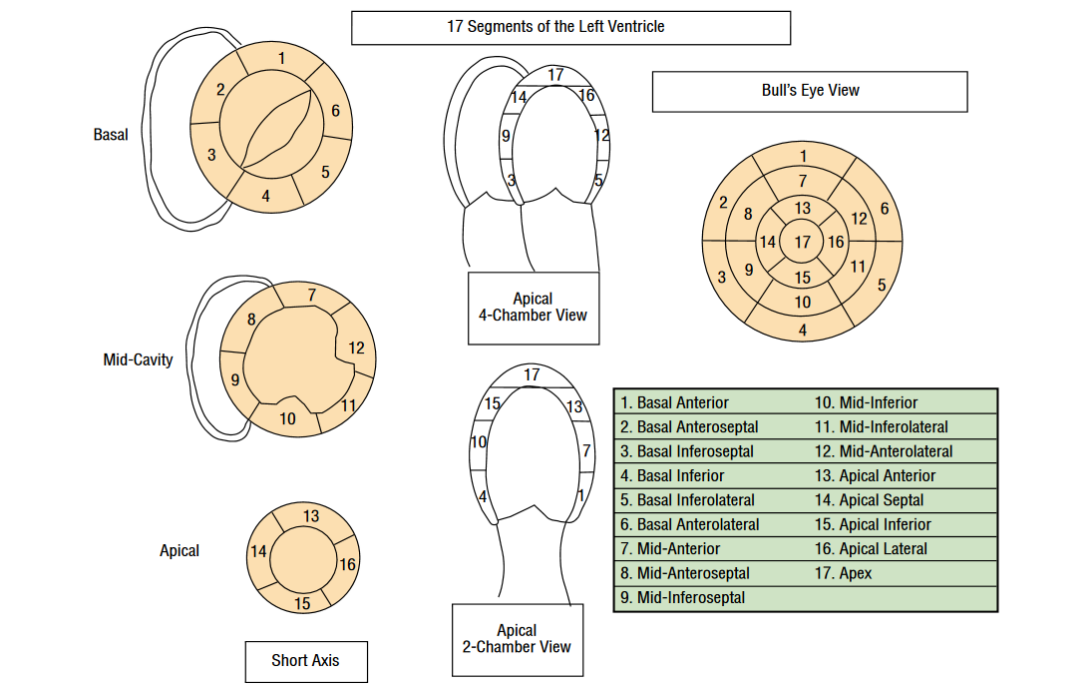
Classification of the 17 segments of the left ventricle in bull’s-eye view, echocardiographic parasternal short-axis slices, and apical 4- and 2-chamber views.

A precise description of the cancer and information about main risk factors of a cardiac event (eg, age, body mass index [BMI], smoking status, blood pressure, hypertension, diabetes, hypercholesterolemia, etc) are collected at inclusion. In addition, we collect information on the use of hormonotherapy, such as antiaromatase, as it may lead to an increased cardiovascular risk [43,44]. Cardiac imaging (ECHO-ST, CT, and MRI) and circulating biomarkers measurements are detailed in Textbox 2 and Figures 1 and 2. Strain measurements will be based on automated ECHO-ST, which partly depends on the type of device (eg, GE, Philips, and Siemens). Not all the centers use the same device, but each patient from each center will be followed using the same device; this will allow comparing the evolution of strain measurements from baseline to the end of follow-up as a relative value, without been biased by the type of device [45].

### Dosimetry

For cardiac dosimetry, automated segmentation of all cardiac structures (including coronary arteries) is performed to ensure the uniformity of the segmentation procedure across centers. This multiatlas automatic segmentation tool of the heart, developed by UMCG, is based on the atlas by Feng et al [48] (Mirada RTx [version 1.6]; Mirada Medical, Oxford, United Kingdom) [49]. This automatic segmentation allows reducing the interobserver variability in contouring organs at risk. With delineated volumes, it is possible to calculate the exact planned radiation dose for the different volumes of the heart. Using the individual doses of patients, the dose-effect relationship can be calculated independently of RT technique or treatment volume. For the cardiac structures such as the whole heart, left atrium, right atrium, left ventricle, right ventricle, strain segments of the left ventricle, and coronary arteries, a minimum dose (*D*_min_) is obtained, as well as a maximum dose (*D*_max_), a mean dose (*D*_mean_), volumes of the structure receiving at least 1, 2, up to the total dose of Gy (*V*_1_, *V*_2_,...until the *V*_total dose_). The same is obtained for the *D*_1_, *D*_2_, and so on, up to *D*_100_ (the dose to 1%, 2%, up to the total of 100% of the volume).

### Study Endpoints

#### Primary Endpoint

The primary endpoint is a mean decrease in the global longitudinal strain or global longitudinal strain rate, determined using cardiac ECHO-ST, of at least 2.5% between the baseline and 24 months after RT [50,51]*.*

#### Secondary Endpoints

Secondary endpoints are defined as follows:

- Changes in myocardial function compared with baseline assessed using echocardiography 6-24 months after RT.
- Anatomical changes in coronary artery atherosclerosis compared with baseline (number of coronary segments containing any plaque or stenosis and calcium score) on CTCA 24 months after RT. The endpoint is defined as ≥15% changes.
- Myocardial changes (morphology, function, tissue characterization by the delayed enhancement, and pre- or postcontrast T1 mapping) compared with baseline on MRI 6-24 months after RT. The endpoint is an increase in the mean precontrast T1 mapping value of, at least, 7%.
- Temporal changes in circulating biomarkers at the end of RT and 6-24 months after RT compared with baseline. The endpoint is a statistically significant increase or decrease in each biomarker between time points.

### Statistical Analysis

#### Sample Size Calculation

The MEDIRAD EARLY HEART study includes 250 women. With 250 patients, this study will have a statistical power of 80% to detect a minimum decrease of 2.5% in global longitudinal strain between baseline measurement and 24 months after RT measurement [14]. The baseline value was derived from the literature: mean global longitudinal strain before RT = −16.5% (SD 2.1%) [52]. In addition, an alpha level of 5% was considered, based on paired tests for comparison with the baseline reference value. Furthermore, the final size was slightly increased to consider the possible loss to follow-up due to death or other reason.

**Main cardiac imaging and circulating biomarkers in the EARLY HEART study.**

Two-dimensional speckle-tracking echocardiography

- Global and segmental longitudinal strain and strain rate (more details on the definition of segments in Figure 2 [46])
- Global and segmental radial strain and strain rate
- Left ventricular ejection fraction using Simpson’s biplane method
- E/A wave ratio: ratio of peak velocity blood flow from gravity in early diastole (the E wave) to peak velocity flow in late diastole caused by atrial contraction (the A wave)
- E/Ea wave ratio: ratio of peak velocity blood flow from gravity in early diastole (the E wave) to Early diastolic velocity of lateral mitral annulus (e’ lateral)
- Tricuspid annular plane systolic excursion
- Heart rate
- Cardiac output measured by multiplying heart rate by the stroke volume

Computed tomography coronary angiography

**Figure 3:**
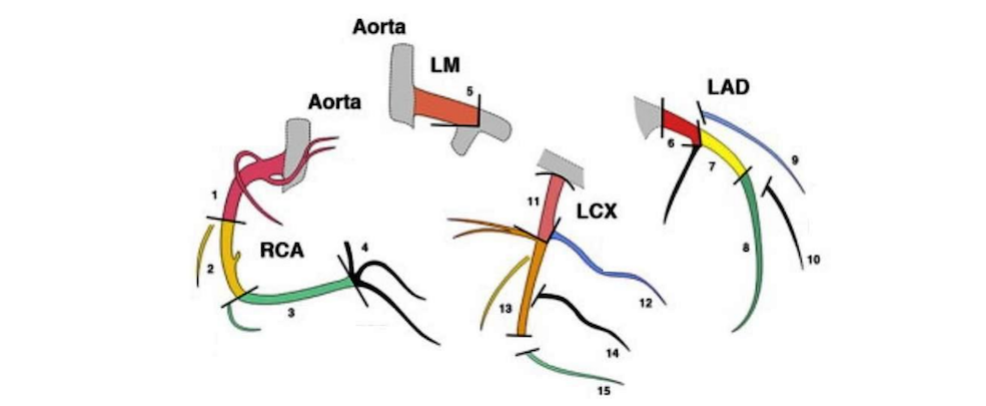
The 15 segments of the coronary arteries. LM: left main coronary; LAD: left anterior descending artery; LCX: left circumflex artery; RCA: right coronary artery.

- Coronary artery calcium score, based on Agatston, volume, and mass; overall and per artery (left main coronary artery, left anterior descending artery, left circumflex artery, and right coronary artery)
- Presence and type of plaque (noncalcified, partly calcified, and calcified); overall and per segment or artery (more details in Figure 3 [47])
- Presence and severity of luminal narrowing based on plaque; overall and per segment or artery

Magnetic resonance imaging

- Cardiac morphology: right ventricular end-diastolic and end-systolic volumes, left ventricular end-diastolic and end-systolic volumes, left ventricular mass, etc
- Cardiac function: left ventricular ejection fraction, right ventricular ejection fraction
- Presence and extent of myocardial infarction based on delayed enhancement
- Tissue characterization based on pre- or postcontrast T1 mapping
- Presence of myocardial edema based on T2 mapping

Circulating biomarkers

- Classical markers:
  - Markers of cardiac injury: C-reactive protein, Troponin I, Troponin T, B-type natriuretic peptide (BNP), N-terminal-proBNP, beta2-microglobulin, Galectin 3

- New biomarkers:
  - Extracellular vesicles: microparticles: CD14 (monocytes), CD31 (endothelial), CD41 (platelets), CD3 (lymphocytes), CD235a (erythrocytes); exosomes
  - MicroRNAs (miRNAs): cardiac miRNAs (miR-1, miR-24, miR-133a/b, miR-208a/b, miR-210); noncardiovascular miRNAs (miR-122)
  - Circulating DNA methylation

#### Planned Analysis

Differences in biomarkers between unexposed and paired exposed groups (eg, right- vs left-sided breast RT) at different time points will be analyzed to generate preliminary hypotheses on the effects of RT on the heart. To investigate the time course of continuous variables extracted from ECHO-ST, CTCA, or MRI measurements and the relation with radiation dose to a variety of cardiac structures, mixed regression models will be used. Confounding variables such as age, blood pressure, BMI, smoking status, hypertension, hypercholesterolemia, or use of hormonotherapy will be included in the risk models. Changes in cardiac biomarkers will be correlated with dose distribution data. An integrative clinical-biological risk score will be developed for individual risk prediction and, finally, multivariable NTCP models will be constructed integrating these biomarkers combined with dose metrics of cardiac structures based on 3D-dosimetry.

All statistical analyses will be performed using SAS statistical software for Windows (SAS Institute, Cary, NC). Furthermore, linear regression models will be performed using generalized estimating equations (Proc Genmod) and linear mixed models (Proc Mixed). An alpha level of .05 will be accepted as significant.

## Results

The inclusion of patients in this European cohort began in 2017. Preliminary results are expected to be published in 2019, and complete analysis should be published in 2021.

## Discussion

### Summary

The MEDIRAD EARLY HEART study is an original multidisciplinary approach to detect and evaluate early radiation-induced cardiotoxicity based on a prospective multicenter cohort study that will finally include 250 patients. It was designed to combine both cardiac imaging information regarding potential early myocardial dysfunction, anatomical coronary changes, and changes in a large panel of circulating cardiac damage biomarkers occurring within first 2 years of RT, based on a precise cardiac dosimetry, allowing to analyze the effect of not only mean heart dose but also doses absorbed by specific heart structures, which better reflect the heterogeneity of dose absorbed by the heart. Provided with patients’ own biological, clinical, and dosimetric parameters, our risk models should allow obtaining individualized risk for patients and enhance knowledge for primary and secondary prevention in these patients.

### Strengths and Limitations

A strength of the MEDIRAD EARLY HEART study is the use of ECHO-ST, CTCA, and MRI, which is the most complete approach used for assessing cardiac changes so far. Effects of specific doses to the whole heart and to specific cardiac substructures have only been assessed in a few studies [8,53] that have revealed the importance of better knowledge for assessing the effects of RT to critical structures of the heart, including the effect of both radiation dose and volume of the heart exposed, further illustrated in a previous study that showed that the use of mean heart dose as a surrogate to the coronary doses was not reliable [47,53,54]. This heart substructure dosimetry was retained for the MEDIRAD EARLY HEART study with an automatic method for the treatment of dosimetry that should allow us to obtain consistent results. The approach of simultaneous assessment of multiple biomarkers, including EVs and cardiac miRNAs, has never been used before and should help understand some biological mechanisms involved in the radiation-induced cardiac changes. The study will allow producing short-term results about subclinical cardiac changes but will not produce, at this stage, results on long-term consequences of early cardiovascular changes. The long-term significance of the observed changes will remain an important issue, and our results should help focus on “at-risk” patients for longer follow-ups.

### Conclusions

In the context of RT, cardiac risks exist due to the presence of normal cardiac tissue in the irradiated field; this can, unfortunately, affect the quality of life of BC survivors, whose numbers are increasing. As a consequence, there is a need for further research to improve the early detection of late cardiac effects in mostly asymptomatic patients and also to improve the prediction and prevention among these patients [55]. MEDIRAD EARLY HEART results will allow for the optimization of RT protocols, leading to personalized treatments with increased therapeutic efficacy and will, therefore, contribute to improve the radiation protection of BC patients. Additionally, it should improve the prediction and prevention of potential lesions to normal cardiac tissues surrounding tumors and ultimately enhance patients’ care and quality of life.

## Abbreviations

BC: breast cancer
BLOOD: blood sample for circulating biomarkers
CAC: coronary artery calcium
CAD: coronary artery disease
CT: computed tomography
CTCA: computed tomography coronary angiography
ECHO-ST: two-dimensional speckle-tracking echocardiography
EV: extracellular vesicle
miRNAs: microRNAs
MRI: magnetic resonance imaging
NTCP: Normal Tissue Complication Probability
RT: radiotherapy
UMCG: University Medical Center Groningen
